# *Symbiodinium* Genotypic and Environmental Controls on Lipids in Reef Building Corals

**DOI:** 10.1371/journal.pone.0020434

**Published:** 2011-05-26

**Authors:** Timothy F. Cooper, Michael Lai, Karin E. Ulstrup, Sandra M. Saunders, Gavin R. Flematti, Ben Radford, Madeleine J. H. van Oppen

**Affiliations:** 1 Australian Institute of Marine Science, UWA Oceans Institute, Crawley, Western Australia, Australia; 2 School of Biomedical, Biomolecular and Chemical Sciences, University of Western Australia, Crawley, Western Australia, Australia; 3 DHI Water and Environment, Perth, Western Australia, Australia; 4 Australian Institute of Marine Science, Townsville, Queensland, Australia; Biodiversity Insitute of Ontario - University of Guelph, Canada

## Abstract

**Background:**

Lipids in reef building corals can be divided into two classes; non-polar storage lipids, e.g. wax esters and triglycerides, and polar structural lipids, e.g. phospholipids and cholesterol. Differences among algal endosymbiont types are known to have important influences on processes including growth and the photobiology of scleractinian corals yet very little is known about the role of symbiont types on lipid energy reserves.

**Methodology/Principal Findings:**

The ratio of storage lipid and structural lipid fractions of Scott Reef corals were determined by thin layer chromatography. The lipid fraction ratio varied with depth and depended on symbiont type harboured by two corals (*Seriatopora hystrix* and *Pachyseris speciosa*). *S. hystrix* colonies associated with *Symbiodinium* C1 or C1/C# at deep depths (>23 m) had lower lipid fraction ratios (i.e. approximately equal parts of storage and structural lipids) than those with *Symbiodinium* D1 in shallow depths (<23 m), which had higher lipid fraction ratios (i.e. approximately double amounts of storage relative to structural lipid). Further, there was a non-linear relationship between the lipid fraction ratio and depth for *S. hystrix* with a modal peak at ∼23 m coinciding with the same depth as the shift from clade D to C types. In contrast, the proportional relationship between the lipid fraction ratio and depth for *P. speciosa*, which exhibited high specificity for *Symbiodinium* C3 like across the depth gradient, was indicative of greater amounts of storage lipids contained in the deep colonies.

**Conclusions/Significance:**

This study has demonstrated that *Symbiodinium* exert significant controls over the quality of coral energy reserves over a large-scale depth gradient. We conclude that the competitive advantages and metabolic costs that arise from flexible associations with divergent symbiont types are offset by energetic trade-offs for the coral host.

## Introduction

Reef building corals (Scleractinia) associate with photosynthetic microalgal endosymbionts from the diverse genus *Symbiodinium* and are therefore considered mixotrophic organisms. Consequently, corals are energetically dependent on carbon assimilation from autotrophy whereby photosynthates of *Symbiodinium* are translocated to the host [1], as well as heterotrophic uptake of carbon such as the capture of zooplankton [2] and the digestion of organic particulate matter [3], [4], [5] by the coral host. Any carbon that is surplus to metabolic requirements is stored as lipids or excreted in mucus [6], [7]. Symbiont identity and diversity is known to exert important influences over the physiology and ultimately fitness in some corals [8], [9]. However, the contribution to lipid stores by distinct *Symbiodinium* types has not been addressed previously.

Total lipid content has been shown to fluctuate in response to seasonal changes in temperature [10], changes in levels of nutrients [11], depth [12], diurnal light variability [13] and turbidity [7]. Given its sensitivity to environmental cues, total lipid content is widely considered a relevant bioindicator of coral condition and resilience to environmental impacts [14]. However, precise measurements of total lipid content are problematic due to the potential for sampling errors inherent with determinations based on gravimetric techniques. Lipids in reef building corals can be divided into two distinct categories; non-polar storage lipids such as wax esters and triglycerides, and polar structural lipids such as phospholipids and cholesterol [10]. Given that seasonal variability in lipid contents are specific to non-polar lipid types [10], the lipid fraction ratio has been proposed as an alternative and potentially more robust bioindicator of coral condition [15], [16]. The lipid fraction ratio, which is the ratio between non-polar and polar lipid types as determined by thin layer chromatography (TLC) [15], also omits the necessity for standardizing to surface area, which can be imprecise.

The nutritional condition of healthy corals is related to a combination of autotrophic and heterotrophic carbon and nutrient assimilation. The relative importance of these two uptake mechanisms vary depending on environmental characteristics such as light and food availability. Furthermore, it has been suggested that the nutritional value of autotrophy alone is limited in the absence of heterotrophic uptake to provide essential nutrients [17]. Nevertheless, in highly oligotrophic environments such as Scott Reef, Western Australia, heterotrophic carbon and nutrient acquisition is considered limited for corals as recent studies have found strong carbon cycling between zooplankton and microbial communities in the water column resulting in limited net carbon loss to benthic communities [18]. Benthic communities in such habitats are, therefore, likely to be adapted to a diet based mainly on the translocation of photosynthates from *Symbiodinium*. Such autotrophic dependency has been directly linked to the lipid content of corals through light availability and hence photosynthetic activity of *Symbiodinium* [13]. Other studies have shown strong links between the type of *Symbiodinium* hosted, where 9 clades (A–I) have been identified containing multiple types within each [19], [20], and the energetics of the holobiont, which may have implications for coral growth and thermal tolerance [8], [21], [22], [23]. However, very little is known about the influence of different symbiont types on the energy reserves of corals *in situ* on coral reefs.

**Figure 1:**
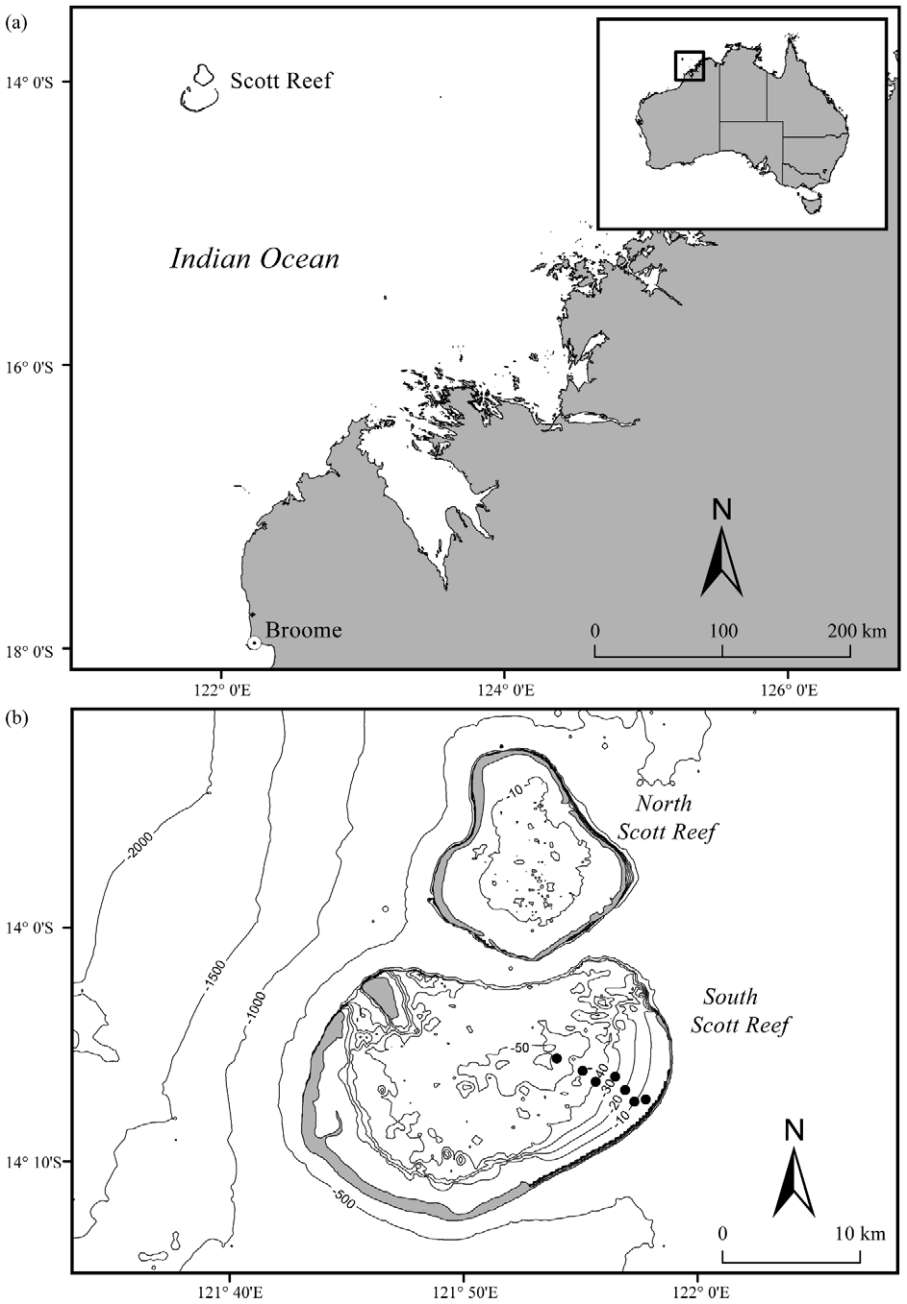
Map of sampling sites of corals collected over a large-scale depth gradient at Scott Reef.

Recent studies at Scott Reef have reported on conspecific corals inhabiting depth gradients of up to 60 m with a distinct zonation pattern in *Symbiodinium* types [24], [25]. Indeed, Cooper et al. (2011) [25] found contrasting metabolic patterns across the depth gradient depending on the *Symbiodinium* consortia hosted. For example, a shift in *Symbiodinium* type of *S. hystrix* at 23 m depth correlated with a shift in the photosynthesis/respiration (P/R) ratio, which increased with depth for colonies associated with clade C types, and similarly those with a clade D type showed a positive response during Spring but negative response with increasing depth during Autumn [25]. These results indicate that the flexibility of *Symbiodinium* types in conspecific corals may exert important controls on the energetic status of the host. The objective of this study was, therefore, to examine the influence of depth and symbiont type on the lipid fraction ratio of *Pachyseris speciosa* and *Seriatopora hystrix* over a depth gradient (3–60 m) incorporating mesophotic depths (i.e. >30 m) at Scott Reef, Western Australia (Figure 1).

## Results

### Environmental setting

There were significant differences in the physico-chemical properties of the water column over the depth gradient at Scott Reef during the study period. Mean concentrations of chlorophyll-*a* were up to four times greater in deep (54 m: 0.58±0.05 µg L^−1^) than shallow water (0 m: 0.14±0.03 µg L^−1^) (Table 1). Mean concentrations of particulate nitrogen, phosphorus, dissolved inorganic nitrogen and dissolved inorganic phosphorus were generally 1.1- to 2-fold greater at deeper depths compared with shallower water (Table 1). Similarly, mean concentrations of particulate organic carbon, dissolved organic nitrogen and dissolved organic phosphorus were between 1.2- and 1.5-fold greater at deeper than shallow depths (Table 1).

**Table 1:** Summary of mean water column parameters (± standard error) along a depth gradient at South Scott Reef, November 2008.

Depth(m)		Chl-*a*(µg L^−1^)	TSS(mg L^−1^)	PN(µmol L^−1^)	PP(µmol L^−1^)	POC(µmol L^−1^)	DIN(µmol L^−1^)	DIP(µmol L^−1^)	DON(µmol L^−1^)	DOP(µmol L^−1^)
0	mean	0.14	0.14	0.65	0.05	11.28	0.84	0.12	10.47	0.47
	se	0.03	0.02	0.07	0.00	1.04	0.18	0.00	0.58	0.16
17	mean	0.37	0.37	0.95	0.07	12.94	0.34	0.11	10.31	0.24
	se	0.06	0.12	0.09	0.00	1.95	0.01	0.00	1.68	0.01
34	mean	0.15	0.84	0.57	0.04	11.81	0.40	0.12	14.79	0.73
	se	0.02	0.41	0.02	0.00	2.84	0.17	0.01	2.10	0.29
45	mean	0.08	0.25	1.00	0.02	11.16	1.16	0.15	11.69	0.58
	se	0.01	0.04	0.00	0.00	2.68	0.24	0.00	0.14	0.20
54	mean	0.58	0.21	0.73	0.06	8.91	1.76	0.17	8.78	0.33
	se	0.05	0.06	0.10	0.00	0.71	0.60	0.01	3.48	0.00

Abbreviations: Chl-*a* = chlorophyll-*a*; TSS = total suspended solids; PN = particulate nitrogen, PP = particulate phosphorus; POC = particulate organic carbon; DIN = dissolved inorganic nitrogen; DIP = dissolved inorganic phosphorus; DON = dissolved organic nitrogen; DOP = dissolved organic phosphorus.

The mean sea water temperature decreased from 30.6±0.04°C at 3 m to 28.2±0.06°C at 50 m. As expected, the light attenuating properties of the water column showed the greatest change over the gradient. Mean midday scalar irradiance decreased exponentially with depth by two orders of magnitude from shallow habitats to the deep lagoon. The mean diffuse attenuation coefficient of scalar irradiance (K_d_ PAR) at the sampling sites was 0.0564 m^2^ indicative of the clear water that is characteristic of this reef.

### *Symbiodinium* associations at Scott Reef

Full details of the analysis of the genetic identity of the *Symbiodinium* assemblage using single-stranded conformation polymorphism (SSCP) procedures in the ITS1 region have been reported previously [25]. In brief, *P. speciosa* hosted predominantly the same *Symbiodinium* C type similar to C3 over the depth gradient sampled. In contrast, *S. hystrix* hosted predominantly *Symbiodinium* D1 at shallow depths between 3–23 m while those in deeper water were dominated by a *Symbiodinium* C type closely related to C1 [25]. The pattern of symbiont zonation in *S. hystrix* has since been confirmed using denaturing gradient gel electrophoresis (DGGE) methodology in the ITS2 region whereby the *Symbiodinium* C type of deeper samples (>23 m) was further resolved as either C1 or C1/C1# [24].

### Lipid fraction ratio

**Figure 2:**
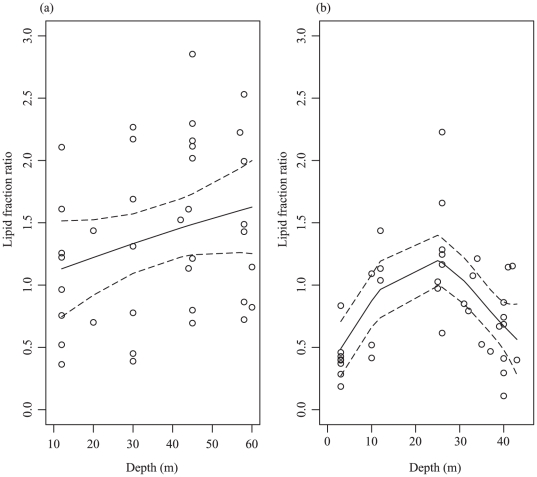
Influence of depth on the lipid fraction ratio of (a) *P. speciosa* and (b) *S. hystrix*. Circles represent data points, smooth line indicates the fitted model and dashed lines are 95% confidence intervals.

Lipid fraction ratio and symbiont genetic identity were determined in 37 samples of *P. speciosa* collected over a depth gradient spanning shallow (12 m) to deep (61 m) depths at South Scott Reef. For *P. speciosa*, the lipid fraction ratio increased linearly from 1.10±0.18 to 1.47±0.22 from shallow to deep depths. Although the lipid fraction ratio of *P. speciosa* showed a weak positive relationship with depth, there was substantial variability between samples collected at the same depth (Figure 2a). Consequently, results of the GAMs showed that neither symbiont type nor depth had a significant influence on the lipid fraction ratio of *P. speciosa* (Table 2a).

**Table 2:** Summary of GAMs investigating the influence of symbiont types and depth on the lipid fraction ratio of *P. speciosa* and *S. hystrix*.

	Estimate	SE	t value	P
*Pachyseris speciosa*				
Symbiont type	0.797	0.666	1.197	0.2400
Depth	1.000	1.000	3.465	0.0713
Symbiont type x Depth	2.000	2.000	2.075	0.1410
*Seriatopora hystrix*				
Symbiont type	0.211	0.101	2.090	**0.0434**
Depth	2.480	3.060	5.559	**0.0030**
Symbiont type x Depth	2.000	2.001	1.670	0.2027

Numbers in bold denote statistical significance α<0.05.

For *S. hystrix*, lipid fraction ratio and symbiont type were determined in 40 samples collected from shallow (3 m) to deep (45 m). In contrast to the pattern observed for *P. speciosa*, there was a non-linear response in the lipid fraction ratio of *S. hystrix* gradually increasing with depth (from <0.50 to >1.50), reaching a peak between 20 and 30 m before decreasing to a value of ∼0.50 at around 45 m (Figure 2b). Importantly, results of the GAMS showed that both symbiont type and depth had significant effects on the lipid fraction ratio of *S. hystrix* (Table 2b).

**Figure 3:**
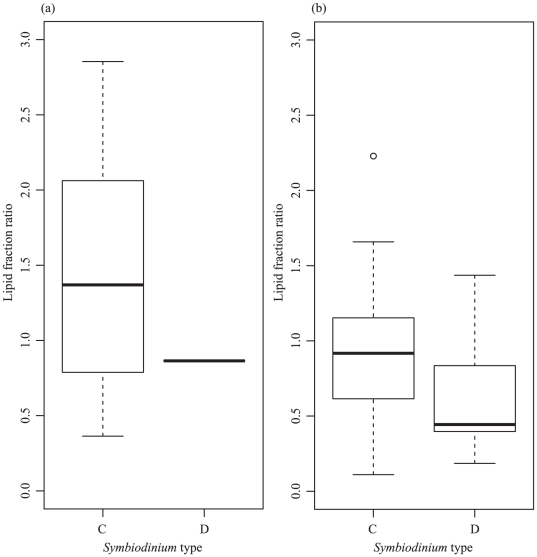
Box and whisker plots of the influence of symbiont type on the lipid fraction ratio of (a) *P. speciosa* and (b) *S. hystrix* at South Scott Reef. For the lines in a box and whisker plot: error bars are the 95% confidence interval, the bottom and top of the box are the 25th and 75th percentiles, the line inside the box is the 50th percentile (median), and any outliers are shown as open circles.

In *P. speciosa*, which showed high specificity to *Symbiodinium* C3, the mean lipid fraction ratio of corals associated with this symbiont type was 1.39±0.10 (Figure 3a). In contrast, *S. hystrix* displayed low symbiont-host specificity where the mean lipid fraction ratio of colonies associated with *Symbiodinium* C1 or C1/C1# and *Symbiodinium* D1 was 0.91±0.08 and 0.58±0.09, respectively (Figure 3b).

## Discussion

### Influence of symbiont genetic identity on coral energy reserves

This study has demonstrated that in addition to changes in other environmental characteristics, *Symbiodinium* may exert significant controls over the quality of the energy reserves of coral hosts over a large-scale depth gradient. Studies of the physiological mechanisms that permit a wide depth distribution of some corals have unveiled the benefits of a flexible relationship with divergent symbiont types to obtain a competitive advantage in habitats with contrasting light regimes [26], [27], [28], [29] and see review by [30]. Most comparative physiological studies of the functioning of *Symbiodinium* have focused on the trade-off between symbiont type and metabolic costs such as effects on growth or photosynthetic performance. For example, Little et al. 2004 [21] demonstrated that juvenile corals associated with *Symbiodinium* C1 had growth rates (defined as the number of polyps per colony) that were two to three-fold greater as well as lowered thermal tolerance than conspecifics harbouring *Symbiodinium* D. This opened the door to the hypothesis that thermal tolerance was provided by divergent symbionts at the expense of growth of the holobiont. Whilst shuffling from thermo-sensitive (i.e. clade C) to thermo-tolerant (clade D) *Symbiodinium* from within the symbiont consortia represents an adaptive strategy to cope with environmental change [9], [31], a recent study has confirmed the hypothesis that shuffling may incur a metabolic cost with growth rates about one third slower in corals packing clade D than those associated with clade C [22]. Similar influences of specific *Symbiodinium* types on the photobiology among different corals have also been recognised [32], [33], [34]. For example, differences in photosynthetic efficiency determined by chlorophyll-*a* fluorescence were observed between two coral species that associated with divergent symbiont types [27]. Similarly, Ulstrup et al. 2011 [35] found that gross photosynthesis rates varied depending on associations between geographically distinct conspecifics with either *Symbiodinium* C or D [35]. At Scott Reef, *P. speciosa* showed high symbiont specificity to a *Symbiodinium* C type similar to C3 over the entire depth gradient. In contrast, *S. hystrix* colonies could be considered infidels due to their association with symbionts belonging to both clades C and D with a shift from *Symbiodinium* D1 to C1 or C1/C1# (a new clade C type; [24]) occurring at a depth of approximately 23 m. This shift in *Symbiodinium* type has previously been correlated with a shift in the photosynthesis/respiration (P/R) ratio of *S. hystrix* albeit for a different sampling time not considered here [25]. Nevertheless, a similar pattern occurred in this study for the energetic reserves of *S. hystrix*, where the shift in symbiont types from *Symbiodinium* D1 to C types (C1 or C1/C1#) was related significantly to the non-linear response in lipid fraction ratio of the coral host, which had a modal peak along the depth gradient at around 23 m. To our knowledge, this is the first report of symbiont type influencing the energy stores of a reef building coral.

### Environmental controls on coral energy reserves

Environmental controls on the lipid fraction ratio of corals sampled at Scott Reef contrasted with the patterns reported elsewhere. Elevated levels of turbidity and low irradiance were correlated with low lipid fraction ratios of *Acropora nobilis* on inshore reefs of North Western Australia [15]. Harland et al. (1992) [12] examined the effect of light and food on storage lipids in the sea anemone *Anemonia viridis* in controlled laboratory experiment. They reported that storage lipids increased with elevated irradiance (experimental light treatments; 0–300 µmol photons m^−2^ s^−1^) after a 60 day exposure. Here, we report contrasting relationships between depth and the lipid fraction ratio for two species of coral. In *S. hystrix* the non-linear relationship was statistically significant whereas *P. speciosa* showed an increase in the lipid fraction ratio, i.e. indicative of a reduction in storage lipids, with increasing depth. The contradiction between our findings particularly for *P. speciosa*, which showed high symbiont specificity, and the experimental results [12] is unclear but could potentially highlight the difference between depth gradients where light is attenuated through the water column and across water quality gradients where differential exposure to nutrients and suspended particulate matter represents a heterotrophic source of nutrition [7].

### Ecological implications of energetic trade-offs among divergent *Symbiodinium* types

The competitive advantages and metabolic costs that arise from flexible associations with divergent symbiont types are offset by energetic trade-offs for the coral host. The proportional relationship between the lipid fraction ratio and depth for *P. speciosa*, which exhibited high symbiont specificity across the depth gradient, was indicative of greater amounts of storage lipids contained in the deep colonies. The reasons for the contrasting pattern between *P. speciosa* (*Symbiodinium* C3) and *S. hystrix* (*Symbiodinium* C1 or C1/C# types) in the response of the lipid fraction ratio at depths beyond 23 m are unclear. Further studies are needed to determine if this is a result of sub-cladal variation in photosynthetic carbon assimilation (*sensu* [34]), depth related differences in the timing of reproduction or some other factor such as differences in the internal harvesting of light in the coral tissue due to morphological differences in the skeletal architecture of the two coral species [36]. Variation in symbiont density may also have contributed to the observed patterns in lipid fraction ratio. Greater symbiont densities in corals from shallow depths correspond to increased amounts of membrane material that could change the amount of polar structural lipids. Indeed, the density of symbionts in *P. speciosa* are known to be higher at shallow depths and decrease with depth at Scott Reef [25], which could explain the patterns in lipid fraction ratio for *P. speciosa*. However, the invariant depth pattern in symbiont density of *S. hystrix* at Scott Reef [25] lends further support to the view of a *Symbiodinium* genotypic control on lipid fraction ratios for this species. Based on the increase in the P/R ratio coupled with the decrease in the lipid fraction ratio of *Symbiodinium* C types harboured by *S. hystrix* over the same depth range, we conclude that the shift in symbiont types in *S. hystrix* is regulated by the trade-off between adaptive strategies to cope with climate change and photoadaptation to low light [26] to remain competitive in deep water habitats.

Metabolic costs manifesting as differences in coral growth rates among different *Symbiodinium* types [21], [22] have been confirmed by differential assimilation rates of carbon through photosynthesis for some *Symbiodinium*-coral associations [23]. Given that corals harbouring *Symbiodinium* C have been shown to assimilate more carbon than *Symbiodinium* D, and that the oceanic environment at Scott Reef is characterised by highly transparent waters with tight carbon pelagic coupling, we hypothesized that deeper *S. hystrix* colonies (those containing *Symbiodinium* C types) would have greater energy stores in the form of storage lipids, and hence a lower lipid fraction ratio, than shallow water (*Symbiodinium* D type) conspecifics. However, this hypothesis was not supported by our results that showed higher lipid fraction ratios for *Symbiodinium* C colonies (i.e. approximately equal parts of storage and structural lipids) than *Symbiodinium* D colonies (i.e. approximately double amounts of storage relative to structural lipid). These results imply that the association with specific symbiont types may be energetically advantageous in the form of an increase in storage lipids relative to structural lipids as well as for coping with climate change\. Clearly, bleaching-tolerant symbionts are needed in shallow water where SST anomalies combined with high light levels may exceed bleaching thresholds, but the metabolic costs associated with them in the form of slower growth [21], [22] and lower P/R ratios [25] are offset by the investment into energy stores. The greater storage lipid reserves in colonies with *Symbiodinium* D could possibly be a strategy to guard against lean times if the symbionts are expelled during a summer bleaching event; analogous to squirrels storing acorns to survive the winter.

This study has provided further insight to the role of *Symbiodinium* in the physiological and metabolic performance of the coral holobiont. The consistency between our patterns in the lipid fraction ratio and those for photosynthetic/respiration ratio documented in a concurrent study [25] provides a deeper understanding into the competitive advantage of flexible symbioses to allow successful colonization of depth generalists across large depth gradients on coral reefs.

## Materials and Methods

### Sampling sites

**Figure 4:**
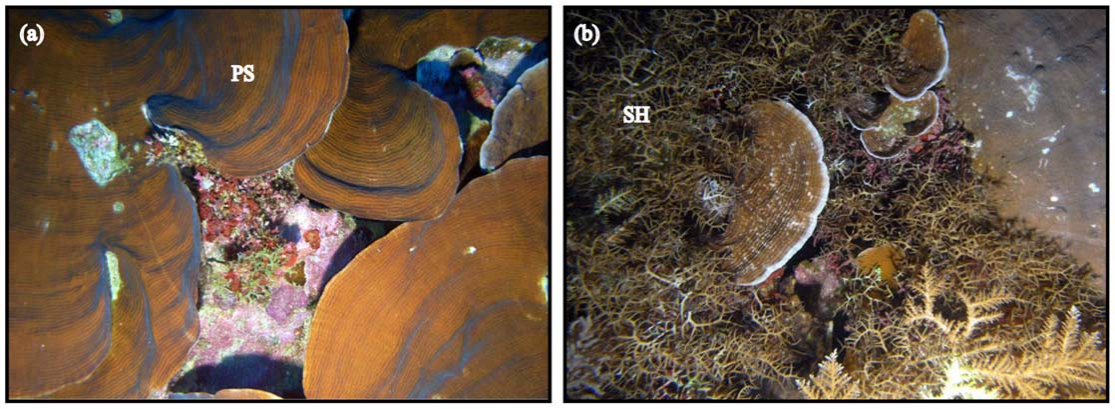
Depth-generalist corals occurring in shallow to deep habitats at Scott Reef. (a) foliaceous *Pachyseris speciosa* (PS) and (b) branching *Seriatopora hystrix* (SH) at approximately 50 m in the central lagoon of South Scott Reef. Images: A. Heyward.

Scott Reef (14°08′S, 121°50′E) is an isolated coral reef system in the Indian Ocean, approximately 270 km from the North West Australian mainland. The foliaceous coral *Pachyseris speciosa* (Agaricidae) and the branching coral *Seriatopora hystrix* (Pocilloporidae) were sampled in November 2008 over a large-scale depth gradient (3–60 m) at South Scott Reef using a combination of snorkelling for shallow samples (3 m) and remote operated vehicle (ROV; LBV 150, Seabotix, Seattle) and video-mounted van Veen benthic grab for deeper samples (Figure 4).

### Genetic analysis

The genetic identity of *Symbiodinium* communities was examined in both coral species sampled at South Scott Reef as described by Cooper et al. (2011) [25]. Briefly, small fragments from each of five replicate colonies of both species were fixed in absolute ethanol for down-stream DNA extraction. Following DNA extraction, zooxanthellae were genetically identified using Single-Stranded Conformation Polymorphism (SSCP) methodology targeting the zooxanthella internal transcribed spacer region (ITS1) [37], [38]. Most samples exhibited SSCP profiles with multiple bands, some of which did not align with SSCP reference samples of known ITS1 sequence. For representatives of each SSCP profile, the zooxanthella ITS1 PCR product was cloned and all cloned bands visible in the original profile were sequenced in order to identify all sequence types present. Standard cloning and sequencing methods were used [38].

### Environmental parameters

Water samples were collected by Niskin bottles (5 L) at five depths (0, 17, 34, 45, 54 m) to represent the depths sampled for determination of genetic identity and lipid fraction ratio along the gradient in the lagoon of South Scott Reef (Figure 1). The water column analyses included concentrations of chlorophyll-*a*, particulate nitrogen, phosphorus and organic carbon, total suspended solids, dissolved inorganic nutrients (NH_4_^+^, NO_2_^−^, NO_3_^−^, PO_4_^2−^), total dissolved nutrients (total dissolved nitrogen [TDN] and total dissolved phosphorus [TDP]) and dissolved organic nutrients (dissolved organic nitrogen [DON] and dissolved organic phosphorus [DOP]). Analytical techniques followed standard procedures [39].

Seawater temperature was measured using a CTD (SBE 19plus V2 SEACAT; Sea-Bird Electronics, Inc, Washington, USA). Two replicate CTD casts were done at each sampling station. Maximal scalar irradiance was determined at midday at each location using an underwater spherical quantum sensor (LI-193, LI-COR, Nebraska, USA) measuring PAR between 400 to 700 nm as described by Cooper et al. (2011) [25].

### Analysis of lipid fraction ratio

Immediately following collection of the coral samples, a small fragment of each replicate colony was placed in liquid nitrogen and stored at −80°C. In the laboratory, all samples were freeze dried for 24 h and stored pending analysis of the lipid fraction ratio. The method of lipid analysis followed techniques described previously [15]. Briefly, five replicates (*n* = 5) of small (∼2 cm) coral samples were weighed, crushed and extracted in chloroform/methanol (2∶1, by volume) using sonication. Following filtration and removal of solvent by evaporation, the mass of lipid obtained was weighed. The lipids extracted were re-dissolved in chloroform/methanol to a constant ratio of 1∶50 (w/v), which was previously shown to maximise grey scale resolution for densitometric measurements [15].

Separation of the total lipid extract into lipid fractions was carried out by thin layer chromatography (TLC). The chromatogram for each plate was made by carrying out elution in a hexane/ether/acetic acid (14∶6∶0.5, by volume) mixture to full height. After drying in air, the plates were immersed in a phosphoric acid/33% acetic acid/sulphuric acid/0.5% copper sulphate (5∶5∶0.5∶90, by volume) solution for 30 s, then re-dried and placed in an oven at 110–115°C for 15 min. The resulting chromatogram was scanned by an image scanner to generate grey scale images. Relative amounts of the different lipid fractions were estimated from the peak grey scale intensity of each lipid band using a densitometric measurement within the UTHSCA image tool (http://ddsdx.uthscsa.edu/dig/).

### Statistical analysis

Generalized additive models (GAMs, [40], [41], [42]) were chosen to analyze the data as they provide a flexible and robust method to model data where non-linear relationships exist between the response variable and one or more explanatory variables. GAMS were constructed for the dependent variable lipid fraction ratio and the independent variables depth and symbiont type for *P. speciosa* and *S. hystrix*. For the independent variables, the appropriate level of smoothing parameter was determined using generalised cross-validation (GCV). The significance values of main effects and interactions of the resulting GAM models are shown in Table 2 and the GAM fits shown in Figure 2. All models were fitted using the function gam (part of the “mgcv” package [43], [44]) in the R computer programming language [45].
